# Antihyperglycemic Effects of *Annona diversifolia* Safford and Its Acyclic Terpenoids: α-Glucosidase and Selective SGLT1 Inhibitiors

**DOI:** 10.3390/molecules25153361

**Published:** 2020-07-24

**Authors:** Miguel Valdés, Fernando Calzada, Jessica Elena Mendieta-Wejebe, Verenice Merlín-Lucas, Claudia Velázquez, Elizabeth Barbosa

**Affiliations:** 1 Instituto Politécnico Nacional, Sección de Estudios de Posgrado e Investigación, Escuela Superior de Medicina, Plan de San Luis y Salvador Díaz Mirón S/N, Col. Casco de Santo Tomás, CP 11340 CDMX, Mexico; jesmenwej@yahoo.com (J.E.M.-W.); rebc78@yahoo.com.mx (E.B.); 2 UMAE Hospital de Especialidades 2° Piso CORSE Centro Médico Nacional Siglo XXI, Instituto Mexicano del Seguro Social, Av. Cuauhtémoc 330, Col. Doctores, CP 06720 CDMX, Mexico; vereniceitzel_merlin@ymail.com; 3 Área académica de Farmacia, Instituto de Ciencias de la Salud, Universidad Autónoma del Estado de Hidalgo, Km 4.5, Carretera Pachuca-Tulancingo, Unidad Universitaria, CP 42076 Pachuca, Hidalgo, Mexico; cvg09@yahoo.com

**Keywords:** diabetes mellitus, antihyperglycemic activity, *Annona diversifolia* Safford

## Abstract

*Annona diversifolia* Safford and two acyclic terpenoids were evaluated to determine their antihyperglycemic activity as potential α-glucosidase and selective SGLT-1 inhibitiors. Ethanolic extract (EEAd), chloroformic (CHCl_3_Fr), ethyl acetate (EtOAcFr), aqueous residual (AcRFr), secondary 5 (Fr5) fractions, farnesal (**1**), and farnesol (**2**) were evaluated on normoglycemic and streptozocin-induced diabetic mice. EEAd, CHCl_3_Fr, Fr5, (**1**) and (**2**) showed antihyperglycemic activity. The potential as α-glucosidase inhibitors of products was evaluated with oral sucrose and lactose tolerance (OSTT and OLTT, respectively) and intestinal sucrose hydrolysis (ISH) tests; the potential as SGLT-1 inhibitors was evaluated using oral glucose tolerance (OGTT), intestinal glucose absorption (IGA), and urinary glucose excretion (UGE) tests. In OSTT and OLTT, all treatments showed significant activity at two and four hours. In ISH, half maximal effective concentrations (CE_50_) of 565, 662 and 590 μg/mL, 682 and 802 μM were calculated, respectively. In OGTT, all treatments showed significant activity at two hours. In IGA, CE_50_ values of 1059, 783 and 539 μg/mL, 1211 and 327 μM were calculated, respectively. In UGE Fr5, (**1**) and (**2**) showed significant reduction of the glucose excreted compared with canagliflozin. These results suggest that the antihyperglycemic activity is mediated by α-glucosidase and selective SGLT-1 inhibition.

## 1. Introduction

Diabetes mellitus (DM) is a group of metabolic diseases characterized by hyperglycemia resulting from defects in insulin secretion, insulin action, or both. Recently, the International Diabetes Federation (IDF) estimated that between 87% and 91% of the population of high-income countries population suffer from type 2 diabetes mellitus (T2D M) [1]. The World Health Organization (WHO) estimated that 8.8% of the world population (425 million people) suffers from DM; however, this number is constantly and alarmingly increasing. The WHO estimated that the number will increase to 9.9% of the population (629 million people) by 2045 [2,3]. The chronic hyperglycemia of diabetes is associated with long-term damage, dysfunction, and failure of different organs, especially the eyes, kidneys, nerves, heart, and blood vessels [4,5]. However, if hyperglycemia levels are properly managed, these serious complications can be delayed or prevented [1,6]. Several drug groups are available for the treatment of DM, grouped according to their mechanism of action because they act on different therapeutic targets. All of them are focused to reduce and control hyperglycemia levels and the associated complications [7]. These drugs are classified into insulin sensitizers (thiazolidinediones and biguanides), secretagogues (sulfonylureas and meglitinides), glucagon-like peptide-1 (GLP-1) analogues, dipeptidyl peptidase-4 (DPP-4) inhibitors, incretinomimetics, α-glucosidase inhibitors and sodium-glucose cotransporter (SGLT) inhibitors [7,8,9,10,11]. α-glucosidase and SGLT inhibitors are useful drugs because they play an important role in the metabolism of carbohydrates consumed in the diet and therefore in the reduction of postprandial hyperglycemia [12].

After food uptake, complex disaccharides like sucrose need to be metabolized by several mechanisms to be absorbed in the small intestine. First, α-glucosidase enzymes are responsible for the hydrolysis of complex oligosaccharides from the diet, such as sucrose to obtain monosaccharides such as glucose and fructose. Once hydrolyzed, monosaccharides can be transported from the small intestine to the bloodstream via a complex mechanism mediated by SGLT1/SLC5A1 cotransporters and glucose transport facilitating systems (GLUTs) [12]. SGLT is a subgroup of the solute carrier group (SLC5), which includes six members that differ in their preferences for sugar binding. The SGLT family uses the electromechanical gradient of sodium to transport sugar molecules against a chemical gradient into cells. Among them, SGLT1 and SGLT2 are the most studied members and are important for glucose homeostasis by absorbing glucose from the diet in the first portions in the small intestine (SGLT1) and by reabsorbing the filtered glucose in the kidney (SGLT2) [11]. The intestinal monosaccharide absorption mediated by SGLT1 implies that one molecule of glucose is cotransported with two Na^+^ ions in an electrogenic process. The transport of Na^+^ and glucose draws osmotically obligated water from the lumen to the blood. Glucose transport is driven by the activity of Na^+^-K^+^-ATPase expressed in the basolateral membrane. The translocation of glucose from the interstitial space to the systemic side is achieved by GLUT2 through facilitated transport [13].

Several drugs are used as α-glucosidase inhibitors; acarbose is the most commonly drug used from this family. Acarbose inhibits α-glucosidases enzymes found in the small intestinal brush border having higher selectivity for sucrose [14]. Inhibition of sucrose hydrolysis prevents carbohydrate absorption because disaccharides are poorly absorbed. Thus, the therapeutic effect after acarbose administration is generated by a lowering of postprandial glucose [15,16]. Notably, this kind of drug only delays and does not prevent the carbohydrate absorption [15]. Regarding SGLT inhibitors, multiple pharmacological tools have been used to determine the potential of SGLT1 inhibition, including phlorizin, canagliflozin, sotagliflozin, LP-925219, KGA-2727, and GSK-1614235. Among these drugs, canagliflozin is an approved competitive SGLT2 inhibitor that is also a low-potency as SGLT1 inhibitor (IC_50_ 155:1) [11,13]. Canagliflozin is an SGLT2 inhibitor that reduces both renal glucose reabsorption and the renal threshold for glucose, with subsequent increases in urinary glucose excretion (UGE) and reduction in plasma glucose levels [17].

Unfortunately, in addition to the therapeutic effects, both families of drugs have side effects. The α-glucosidase inhibitors can generate mild stomach pain, gas, or stomach bloating, whereas the SGLT inhibitors can produce bladder pain, difficult or painful urination, and a frequent urge to urinate [18,19]. Due to the side effects, there is a need to search for new antidiabetic drugs devoid of side effects to improve the quality of life of the patient. Medicinal plants constitute an important source of new compounds with potential therapeutic effects [20,21,22,23,24]. Multiple plants from traditional medicine are used by as important antidiabetic remedies [25,26,27]. Among these, several species of the Annonaceae family are commonly used for the treatment of DM, such as *Annona squamosa*, *Annona muricata*, *Annona cherimola*, *Annona crassiflora*, *Annona purpurea*, *Annona reticulata*, and *Annona diversifolia* [28,29,30,31,32,33,34,35,36].

*Annona diversifolia* Safford is a common tree indigenous in Mexico known by many local names such as “Ilama”, “Ilama Zapote”, “Ilamazapotl”, “Izlama”, “Hilama”, “Papauce”, “Papausa”, “Anona blanca”, and “Zapote de vieja” [36,37,38,39,40,41]. The fruit of this plant is used as food; its leaves are employed as anticonvulsant, analgesic, anti-inflammatory, and antidiabetic agents in Mexican traditional medicine [37,38,39]. Several pharmacological studies reported its anxiolytic [37], antilymphoma [38], antinociceptive [39], anticonvulsant [40,41], antimicrobial [42], antiproliferative [43], antiepileptic [44], anxiolytic [45], and antihyperglycemic effects [36,46]. The ethanolic extract from the leaves of *A. diversifolia* and farnesol, an acyclic sesquiterpene isolated from this plant, showed antihyperglycemic activity in female mice [36]. In this context, more studies are needed on the antihyperglycemic activity of *A. diversifolia* in male mice, as well as the existence of more products that can be isolated, characterized, and evaluated as potential antihyperglycemic agents, elucidating their mechanism of action.

In this work, we explored the antihyperglycemic activity of the ethanol extract of leaves from *A. diversifolia* (EEAd), chloroformic (CHCl_3_Fr), ethyl acetate (EtOAcFr), aqueous residual (FrAcR), secondary 5 (Fr5) fractions and, in addition to the farnesol (**2**) previously isolated from this plant, another acyclic terpenoid named farnesal (**1**). The products were evaluated as α-glucosidase or SGLT1 inhibitiors. The effects on blood glucose levels were tested in male normoglycemic and streptozocin-induced diabetes type 2 mice (SID2). The compounds with significative activity in SID2 mice were evaluated using oral sucrose, lactose, and glucose tolerance tests (OSTT, OLTT, and OGTT, respectively), and ex vivo assays including the intestinal sucrose hydrolysis inhibition (ISH) and intestinal glucose absorption inhibition (IGA) tests. To evaluate the SGLT-1 selectivity, urinary glucose excretion (UGE) tests was conducted. 

## 2. Results

### 2.1. In Vivo Assays

#### 2.1.1. Acute Antihyperglycemic Activity of Ethanolic Extract from *A. diversifolia* and Its Products

The acute antihyperglycemic activity was evaluated to determine the potential antidiabetic effect of EEAd and its products obtained from *A. diversifolia*. First, all products were evaluated on male normoglycemic mice (NM; Table 1). The groups treated with EEAd, CHCl_3_Fr, Fr5, farnesol, and/or farnesal did not produce a significant decreased in the blood glucose values. In contrast, the groups treated with AcRFr and EtOAcFr showed a significant increase in the blood glucose levels at two and four hours. With respect to antidiabetic drugs canagligflozin, glibenclamide, and pioglitazone, they produced a significant decrease at two and four hours of treatment; the hypoglycemic effect of these treatments is a consequence of their mechanism of action [7].

When the products were tested on male streptozocin-induced type 2 (SID2), EEAd produced a significant decrease in blood glucose levels at two hours of treatment that returned to hyperglycemic values at four hours. In the case of the fractions, CHCl_3_Fr produced a significant decrease in blood glucose levels at two and four hours. In contrast, AcRFr and EtOAcFr, as observed in male NM, produced a significant increase in blood glucose levels; thus, both were excluded from further study. The Fr5 and farnesal showed a significant decrease in blood glucose levels at two and four hours. Farnesol produced a significant decrease in blood glucose levels at two hours that returned to hyperglycemic values at four hours; this effect was similar to that of EEAd. The antidiabetic drugs produced a significant decrease in blood glucose levels at two and four hours (Table 1).

Once the acute antihyperglycemic activity of EEAd, CHCl_3_Fr, Fr5, farnesal, and farnesol were observed, we focused on evaluating their activity as α-glucosidase and selective SGLT-1 inhibitors.

#### 2.1.2. Oral Sucrose and Lactose Tolerance Test of Ethanolic Extract from *A. diversifolia* and Its Products

**Figure 1:**
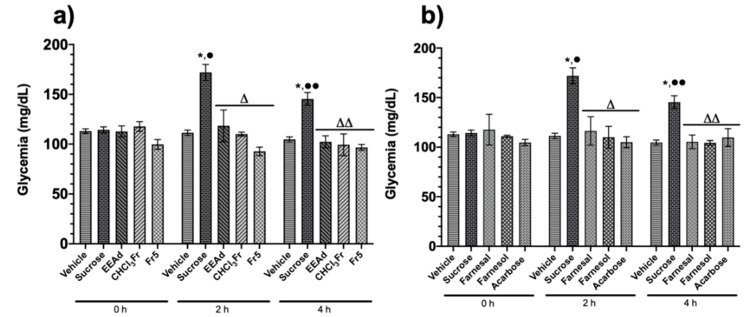
Effect of products isolated from *Annona diversifolia* and acarbose on the oral sucrose tolerance test (OSTT). (**a**) Groups treated with vehicle, sucrose (3 g/kg), EEAd, CHCl_3_Fr (200 mg/kg), and Fr5 (50 mg/kg). (**b**) Groups treated with vehicle, sucrose (3 g/kg), farnesal, farnesol, and acarbose (50 mg/kg). Data are expressed as means ± SEM, *n* = 6; * *p* < 0.05 vs. initial values; ^•^ *p* < 0.05 vs. vehicle for 2 h; ^••^ *p* < 0.05 vs. vehicle for 4 h; ^Δ^ *p* < 0.05 vs. sucrose for 2 h; ^ΔΔ^ *p* < 0.05 vs. sucrose for 4 h.

After the administration of EEAd, CHCl_3_Fr, Fr5, farnesal, farnesol, or acarbose, all treatments showed a significant reduction in the glycemic postprandial peak at two and four hours with respect to the sucrose group (Figure 1). 

**Figure 2:**
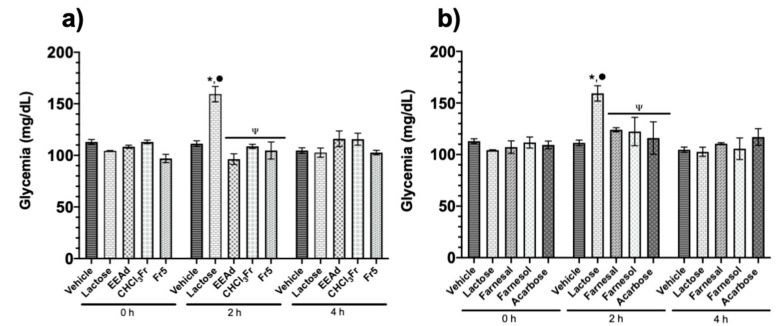
Effect of products isolated from *A. diversifolia* and acarbose on the oral lactose tolerance test (OLTT). (**a**) Groups treated with vehicle, lactose (3 g/kg), EEAd, CHCl_3_Fr (200 mg/kg), and Fr5 (50 mg/kg). (**b**) Groups treated with vehicle, lactose (3 g/kg), farnesal, farnesol, and acarbose (50 mg/kg). Data are expressed as means ± SEM, *n* = 6; * *p* < 0.05 vs. initial values; ^•^ *p* < 0.05 vs. vehicle for 2 h; ^Ψ^ *p* < 0.05 vs. lactose for 2 h.

In the OLTT after administration, all treatments produced a significant reduction in the glycemic postprandial peak at two hours with respect to the lactose group (Figure 2).

The results obtained in the OSTT and OLTT suggested that the antihyperglycemic activity of the products isolated from *A. diversifolia* is mediated by the inhibition of the hydrolysis of complex carbohydrates with glycosidic bond types α-1,4 and β-1,4; this hypothesis was corroborated with ex vivo assays.

#### 2.1.3. Oral Glucose Tolerance Test of Ethanolic Extract from *A. diversifolia* and Its Products

**Figure 3:**
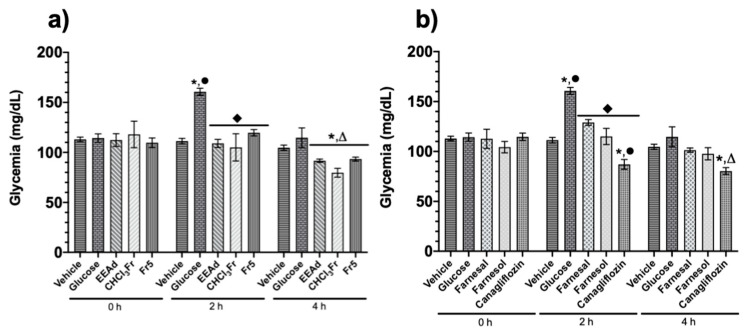
Effect of products isolated from *A. diversifolia* and canagliflozin on the oral glucose tolerance test (OGTT). (**a**) Groups treated with vehicle, glucose (1.5 g/kg), EEAd, CHCl_3_Fr (200 mg/kg), and Fr5 (50 mg/kg). (**b**) Groups treated with vehicle, glucose (1.5 g/kg), farnesal, farnesol, and canagliflozin (50 mg/kg). Data are expressed as means ± SEM, *n* = 6; * *p* < 0.05 vs. initial values; ^•^ *p* < 0.05 vs. vehicle for 2 h; ^⬪^ *p* < 0.05 vs. glucose for 4 h; ^Δ^ *p* < 0.05 vs. vehicle for 4 h.

In the OGTT, after the administration of EEAd, CHCl_3_Fr, Fr5, farnesal, farnesol, or canagliflozin, all treatments produced significant reductions in the postprandial peak at two hours with respect to glucose group at two hours. The EEAd, CHCl_3_Fr, Fr5, and canagliflozin groups showed a significant decrease in blood glucose levels in comparison with vehicle group at four hours (Figure 3).

Next, ex vivo assays were conducted to confirm the inhibition of the hydrolysis of complex carbohydrates mediated by α-glucosidases enzymes, as well as the inhibition of simple carbohydrates absorption mediated by SGLT-1.

### 2.2. Ex Vivo Assays

#### 2.2.1. Intestinal Sucrose Hydrolysis Inhibition Test of Ethanolic Extract from *A. diversifolia* and Its Products

After the addition of EEAd, CHCl_3_Fr, Fr5, farnesal, farnesol, or acarbose, EEAd produced a significant decrease in the quantity of glucose measured in the external aqueous medium (EAM) at 200, 400 and 800 µg/mL compared to the sucrose control with a calculated half maximal effective concentration (CE_50_) of 565.6 µg/mL. CHCl_3_Fr and Fr5 produced a significant decrease at 400 and 800 µg/mL with a CE_50_ of 662.2 and 590.4 µg/mL, respectively. Farnesal and farnesol showed significant decreases at 800 µM with a calculated CE_50_ of 682.9 and 802.2 µM, respectively. Acarbose produced a significant decrease at 200, 400 and 800 µM, with a calculated CE_50_ of 187.82 µM (Table 2).

#### 2.2.2. Intestinal Glucose Absorption Inhibition Test of Ethanolic Extract from *A. diversifolia* and Its Products

After the addition of EEAd, CHCl_3_Fr, Fr5, farnesal, farnesol, or canagliflozin, EEAd significantly decreased the quantity of glucose absorbed in the small intestine (SI) at 800 µg/mL with a calculated CE_50_ of 1059.9 µg/mL. CHCl_3_Fr and Fr5 produced a significant decrease at 400 and 800 µg/mL with a calculated CE_50_ of 783.5 and 539.91 µg/mL, respectively. Farnesal produced a significant decrease at 800 µM with a calculated CE_50_ of 682.8 µM. Farnesol produced a significant decrease at 400 and 800 µM with a calculated CE_50_ of 372.3 µM. Canagliflozin showed significant decreases at 400 and 800 µM, with a calculated CE_50_ of 763 µM (Table 3).

### 2.3. Urinary Glucose Excretion Assay of Ethanolic Extract from A. diversifolia and Its Products

**Figure 4:**
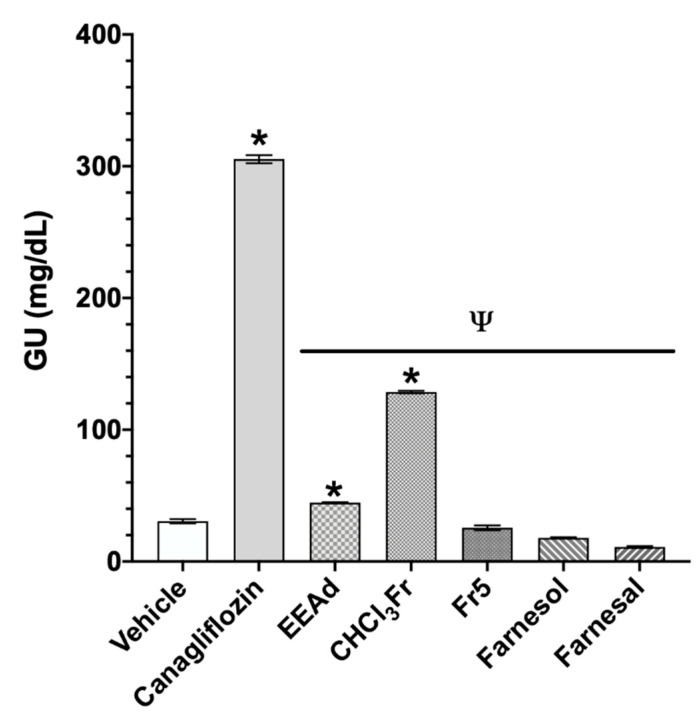
Effect on urinary glucose concentration after administration of products isolated from *A. diversifolia* and canagliflozin on the urinary glucose excretion (UGE) assay. Data are expressed as means ± SEM, *n* = 6; * *p* < 0.05 vs. vehicle; ^Ψ^ *p* < 0.05 vs. canagliflozin. GU: glucose in urine.

In the UGE assay, after administration of the antidiabetic drug canagliflozin used as control, we observed a significant increase in the urinary glucose concentration in comparison with the vehicle group. The groups treated with EEAd and CHCl_3_Fr showed significant increases in the urinary glucose concentration in comparison with vehicle group. EEAd, CHCl_3_Fr, Fr5, farnesol, and farnesal groups showed significant lower values in comparison with the canagliflozin group (Figure 4).

## 3. Discussion

Diabetes mellitus is a serious chronic disease worldwide. Oral antihyperglycemic standard medication is often successful in the treatment of this disease; however, all of them have several side effects [47]. One of the principal approaches for reducing postprandial hyperglycemia in patients with DM is the prevention of hydrolysis and the absorption of carbohydrates after food uptake due to the effective control of blood glucose levels being a key step in preventing or reversing diabetic complications and improving the quality of life in diabetic patients [7,12].

We aimed to evaluate the antihyperglycemic activity of the ethanol extract of leaves from *A. diversifolia* (EEAd), the CHCl_3_Fr and Fr5 fractions, and two compounds isolated (farnesal and farnesol) using the activity-guided fractionation as a strategy, as well to evaluate the possible mechanism of action of the antihyperglycemic products as α-glucosidase or SGLT1 inhibitors. Results from the tests indicated that the control of postprandial glucose levels shown by the products from *A. diversifolia* might be due to an antihyperglycemic effect mediated by the regulation of glucose uptake from the intestinal lumen through the inhibition of complex carbohydrate digestion (OSTT, OLTT, and SIH) or simple carbohydrate absorption (OGTT and IGA). The reduction of the postprandial peak after a complex carbohydrate load (sucrose) or a simple carbohydrate (such as glucose) may be associated with the inhibition of intestinal α-glucosidase (OSTT, OLTT, and SIH) or the selective inhibition of SGLT1 (OGTT, IGA and UGE) [12,47,48].

First, EEAd was evaluated in NM and SID2 male mice and the results demonstrated the antihyperglycemic activity of the extract; notably, this effect is labeled as such because the administration of the extract does not generate hypoglycemia in NM animals. In accordance with studies conducted in other species of the Annonaceae family, the genus was shown to possess antihyperglycemic activity [28,33]. Comparing our results with those of previous studies, the antihyperglycemic activity demonstrated by EEAd is effective in female and male BALB/c mice. This antihyperglycemic activity was corroborated at a dose of 200 mg/kg in both sexes [36]. 

The next step in the activity-guided fractionation was to evaluate the fractions obtained from *A. diversifolia* (CHCl_3_Fr, AcRFr, EtOAcFr, and Fr5). The results showed that CHCl_3_Fr was the fraction with antihyperglycemic activity in SID2 mice and did not generate hypoglycemia in NM. AcRFr and EtOAcFr both generated hyperglycemia in male SID2 and NM mice; thus, both were excluded from future study. Compared with our study, antidiabetic evaluations in other species of the Annonaceae family showed that the polar fraction is principally responsible for the activity, with rutin, quercetin, isoquercetrin, astragalin, kaempferol, among other flavonoids, being described as the products responsible for the antihyperglycemic activity [32,33,34,41]. The presence of any of these flavonoids in EEAd was not ruled out; however, it is possible that there was a higher concentration of carbohydrates such as sucrose in FrAcR and FrEtOAc that favored the increase in blood glucose in the animals [41].

**Figure 5:**
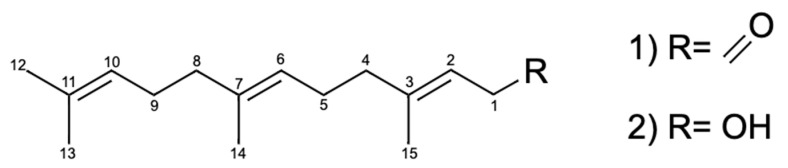
Structures of the compounds farnesal (**1**) and farnesol (**2**) isolated from *A. diversifolia*.

The Fr5 obtained from CHCl_3_Fr showed considerable antihyperglycemic activity at a dose of 50 mg/kg. This result agrees with that of a previously study and confirmed that this fraction is one of the products responsible of the antihyperglycemic activity of *A. diversifolia* [36]. From Fr5, compounds (**1)** and (**2**) were isolated; these were structurally characterized by spectrometric (MS-EI^+^) and spectroscopic (IR, ^1^H, and ^13^C NMR) methods (Table 4) as farnesal and farnesol, respectively (Figure 5). The evaluation of farnesal and farnesol showed that both have antihyperglycemic activity at 50 mg/kg. The activity showed by EEAd, CHCl_3_Fr, Fr5, farnesal, and farnesol is comparable with the activity demonstrated by acarbose, an α-glucosidase inhibitor currently used in therapy [10]. With respect to antidiabetic drugs, the significant decrease in blood glucose levels at two and four hours showed by glibenclamide suggested that functional β-cells still exist in the SID2 model, and the evidence of a significant decrease of hyperglycemic values after administration of secretagogues suggested that no insulin resistance has yet developed in the SID2 model [12,33].

Next, two mechanisms of action, as α-glucosidase and SGLT-1 inhibitors, were evaluated, considering the main objectives of reducing hyperglycemia in DM patients is the prevention of carbohydrate hydrolysis (α-glucosidase) and absorption (SGLT-1) after food intake [49], and considering that the products contact the stomach and small intestine with oral administration, occurring where the α-glucosidases and SGLT-1 cotransporters function for the hydrolysis and absorption of carbohydrates [12,13]. As several species of the Annonaceae family have been described as potential α-glucosidase inhibitors [30,32,33,36], using a chemotaxonomic criterion, both mechanisms of action were selected.

The in vivo α-glucosidase inhibition was evaluated using OSTT and OLTT [22,33]. In both tests, we observed a significant reduction in the glucose postprandial peak after the administration of EEAd, CHCl_3_Fr, Fr5, farnesol, and farnesal; thus, these results confirmed the antihyperglycemic activity of the products isolated from *A. diversifolia* [36]. The activities demonstrated by all the products were similar to that observed with the antidiabetic drug acarbose, and α-glucosidase inhibitor we used as a control [10]. In vivo SGLT-1 inhibition was evaluated using the OGTT [50]. In this test, we observed that all the treatments reduced the glucose postprandial peak after a monosaccharide load. This may have occurred due to the inhibition of the absorption mediated by SGLT-1 cotransporters [11]; however, more studies are required to verify this theory. Notably, EEAd, CHCl_3_Fr, and Fr5 showed significant reductions in glycemia values four hours after treatment; this can be explained due to the accumulation of glucose in the small intestine, which can generate an increase in the secretion of incretins and insulin, in turn producing a reduction on the glycemia values. This effect added to the inhibition of the glucose absorption, explaining the hypoglycemia [51]. Moreover, it is possible that the products evaluated can improve the insulin signaling, like a thiazolidinedione or biguanide, increasing the glucose utilization in tissues [8,9]. In the group treated with the pharmacological control canagliflozin, the hypoglycemia observed was a result of the non-selective mechanism of action for this drug. In addition to preventing intestinal glucose absorption, canagliflozin inhibits SGLT-2, reducing the renal glucose absorption, thus increasing the UGE [17].

Some authors have described that oral administration of α-glucosidase inhibitor drugs such as acarbose or miglitol could improve insulin sensitivity on insulin-resistant animal models [52,53], this can be associated with thiazolidinedione or biguanide mechanism of action. Thus it is possible that the antihyperglycemic activity shown by the products of *A. diversifolia* also may be related to their capacity of acting as insulin sensitizers, as thiazolidinedione or biguanide, however, more studies are required to evaluate these activities, due to insulin correct utilization is one of the important indicators for monitoring the progression of diabetes mellitus.

In addition to the in vivo OSTT and OLTT, to demonstrate the α-glucosidase inhibitory activity, ex vivo assays were conducted to confirm the reduction of the hydrolysis of complex disaccharides in the small intestine. The ISH inhibition test was conducted using portions of small intestine from Sprague Dawley rats; in these portions of intestine, α-glucosidase enzymes are responsible for hydrolyzing complex disaccharides such as lactose and sucrose into simple monosaccharides to be absorbed [50]. If a complex disaccharide such as sucrose is added, the enzymes present in the small intestine portions are responsible for hydrolyzing the disaccharides, and, subsequently, the monosaccharides resulting from the hydrolysis will be absorbed and can be measured in the external aqueous medium. Thus, by adding an α-glucosidase inhibitor such as acarbose, the decrease in the amount of glucose in the external medium can be interpreted as a result of the inhibition of α-glucosidase. After the addition of the products, EEAd showed significant activity with respect to sucrose control at 200, 400 and 800 µg/mL. CHCl_3_Fr and Fr5 showed significant activity at 400 and 800 µg/mL. These results can be explained by EEAd containing a larger amount of compounds including high polarity compounds such as flavonoids that have been described as α-glucosidase inhibitors [32,33,34,41]. Farnesal and farnesol only showed significant activity at 800 µM with a CE_50_ of 682 and 802 µM, respectively. These results confirmed the reports in the literature of both molecules as possible α-glucosidase inhibitors [12].

To corroborate the OGTT activity, ex vivo IGA inhibition tests were conducted considering that the monosaccharides can be transported, once hydrolyzed, from the small intestine into the bloodstream through a complex mechanism mediated by SGLT-1 cotransporters [17]. The IGA test applies the same concept as the ISH assay, but in this case, we used a simple carbohydrate such as glucose, since it does not need to be hydrolyzed, the absorption is instant, and it can be measured in the external aqueous medium. Thus, if a SGLT-1 inhibitor such as canagliflozin is added in the small intestine, the decrease in the amount of glucose in the external aqueous medium can be interpreted as a result of the inhibition of SGLT-1. After the addition of EEAd, we observed significant activity in comparison to the glucose control at 800 µg/mL with a CE_50_ of 1059 µg/mL. This result agrees with the observed ISH, OGTT and OLTT results. The existence of high polarity compounds in EEAd may have given the extract higher activity in terms of complex carbohydrate hydrolysis than in simple carbohydrate absorption. This result can be observed in the CE_50_ calculated for both tests. However, in vivo assays showed that a dose of 200 mg/kg was enough to reduce hyperglycemia values.

CHCl_3_Fr and Fr5 showed activity at 400 and 800 µg/mL. This result agrees with the observed in OGTT for the fractions and EEAd, and partly explains the hypoglycemia that can be generated by an accumulation of glucose in the small intestine that produces an increase in the secretion of incretins and insulin, which acts to reduce the blood glucose levels. Farnesal produced significant activity at 800 µM; the calculated CE_50_ demonstrated that the antihyperglycemic activity of this compound is mediated, to a large extent, by the inhibition of the hydrolysis of complex carbohydrates by α-glucosidases enzymes; however, SGLT-1 activity was not ruled out for farnesal. Farnesol and canagliflozin produced significant activity at 400 and 800 µM; however, the calculated CE_50_ values were 372 and 763, respectively. This result indicated that farnesol has greater activity on the glucose absorption in the small intestine. This can be explained by canagliflozin being a non-selective SGLT-1 inhibitor, having more affinity to SGLT-2 in the kidney [11].

Finally, the SGLT-1 selectivity was investigated using the UGE test. This test showed the possible selectivity of the treatments evaluated toward SGLT-1 inhibition as canagliflozin is an inhibitor with greater selectivity toward SGLT-2 [11]. Therefore, when animals are treated with canagliflozin, they excrete glucose in their urine [50]. The results showed the selectivity of canagliflozin toward SGLT-2 inhibition, which was observed in the significative increase in renal glucose excretion by the group treated with this drug in comparison with the group treated with the vehicle. The same result was observed by the groups treated with EEAd and CHCl_3_Fr, which can be explained due to the large number of compounds that we suggest that may also be acting on SGLT-2 in EEAd and CHCl_3_Fr. In Fr5, farnesal, and farnesol, selectivity toward SGLT-1 is suggested due to the lack of increase in renal glucose excretion after the administration of the products; however, we recommend in silico studies such as molecular docking experiments to determine the potential binding mode at the molecular level of farnesal and farnesol on α-glucosidase and SGLT-1 cotransporter, as well as to evaluate the changes in protein expression or increasing RNA levels to corroborate the results obtained in this study and confirm the mechanism of action. 

These results provide information about the possible mechanisms of action of farnesal and farnesol, confirming their antihyperglycemic activity mediated by the inhibition of α-glucosidase and a selective inhibition of SGLT-1. The results reported from this study for farnesal and farnesol provide a starting point for the development of new drugs for the treatment of DM. The findings indicate that the ethanolic extract obtained from the leaves of *A. diversifolia* is effective in vivo for controlling fasting and postprandial blood glucose levels in animal models of diabetes mellitus; thus, leaves from *A. diversifolia* represent a good phytotherapeutic agent for the treatment of this disease. Some advantages include the majority of the Mexican population being able to access this natural remedy and the lower cost of this treatment, providing a cheaper alternative for the treatment of diabetes mellitus.

## 4. Materials and Methods

### 4.1. General Information

Ethanol anhydrous (Catalogue code: 15568604), ethyl acetate (CC:10382681), chloroform (CC: 15508564), and dichloromethane (CC:15594055) solvents were purchased from J.T. Baker™ (Thermo Fisher Scientific, Waltham, MA, USA). Farnesol (95%, CC: F203-25G), farnesal (≥85%, mixture of isomers, CC:46188-1ML-F), streptozocin (≥75% α-anomer basis, PN: S0130-5G ), nicotinamide (≥99.5%, PN: 47865-U), glucose (anhydrous, PN: D9434-1Kg), sucrose (≥99.5% GC, PN: S9378-1Kg), acarbose (PN: PHR1253-500MG), canagliflozin (95%, PN: 721174-1G ), and glibenclamide (PN: PHR1287-1G) were purchased from Sigma-Aldrich^®^ (Sigma^®^, Saint Louis, MO, USA). Buffer solution (citric acid/sodium hydroxide/hydrogen chloride, pH 4.00, CC: 109445), silica gel high-purity grade (7734) pore size 60 Å, 70,230 mesh (CC: 391484-5KG), TLC glass plates L × W 20 cm × 20 cm, sílica gel 60 F_254_, 2 mm (CC: Z292974) were purchased from Merck^®^ (Merck^®^, Darmstadt, Germany). Saline solution 0.9% (solution 1000 mL) and DX-5 glucose solution 5% (solution 500 mL) were purchased from PISA^®^ Pharmaceutics (PISA^®^, Mexico City, México). IR Spectrometer, Model: Tensor-27, Bruker^®^ (Bruker^®^, Billerica, MA, USA); NMR Spectrometer, Model: Avance III, 400 MHz, Bruker^®^ (Bruker^®^, Billerica, MA, USA).

### 4.2. Plant Materials

The leaves of *A. diversifolia* were collected by chemical engineer Jorge Ebrard Maure in Metapa de Domínguez (14°50′00″ N, 92°11′00″ W) in Chiapas, México. The plant material was identified by an in-house botanist (M.Sc. Santiago Xolapa) of the Herbarium of the Instituto Mexicano del Seguro Social (IMSS), with a voucher specimen of 16248. The sample was cleaned of any impurities before being air-dried at ambient temperature until a constant weight was achieved. The dried samples were ground using a laboratory grinder (model M-22-RW, Fundicion Torrey, Apodaca, Nuevo León, México). 

### 4.3. Extraction, Isolation and Identification of Farnesol and Farnesal

The finely grounded leaves of *A. diversifolia* (3 kg) were extracted by maceration at room temperature with EtOH (17 L × 3). The macerated extraction was filtered using filter paper. The filtered extract was collected and concentrated using a rotary evaporator (Büchi Labortechnik AG, Flawil, Switzerland) under vacuum at 40 °C to obtain 175 g of dried extract (EEAd, 5.86% yield). Once we examined the antihyperglycemic activity of EEAd, it was fractionated. Briefly, a portion of EEAd (47.5 g) was suspended in 10% EtOH-water (100 mL) and successively partitioned with CHCl_3_ (100 mL × 3) to obtain 43.31 g CHCl_3_ fraction (CHCl_3_Fr) and EtOAc (100 mL × 3) to obtain 1.69 g of EtOAc fraction (EtOAcFr). The aqueous residual layer was collected to obtain 2.5 g of aqueous residual fraction (AcRFr). The antihyperglycemic activity was associated with CHCl_3_Fr, then a portion (25 g) was subjected to a silica gel chromatography column using 172 g of silica gel and eluted with dichloromethane (DCM) to obtain five fractions: Fr1 (450 mg), Fr2 (210 mg), Fr3 (1380 mg), Fr4 (150 mg), and Fr5 (3000 mg). The most active fraction was Fr5. Then, 800 mg was purified by preparative thin layer chromatography using DCM (100%) to obtain compound (**1**) (31.6 mg) and compound (**2**) (306 mg).

Farnesal (**1**): A yellowish oil, IR (KBr) *v_max_* 3441.22, 2876.32, 2930.7, 2917.2 cm^−1^; MS (EI^+^) *m/z* 220.18312 (calcd for C_15_H _24_O, 220.18271); ^1^H- and ^13^C-NMR (CDCl_3_) see Table 4, Figure 5.

Farnesol (**2**): A greenish oil, IR (KBr) *V_max_* 3531.35, 2964.65, 2930.71, 2875.32 cm^−1^; MS (FAB^+^) *m/z 245* [M + Na^+^] (calcd for C_15_H _26_O, 222.19836) ^1^H- and ^13^C-NMR (CDCl_3_) see Table 4, Figure 5. 

Additionally, both products were subjected to direct comparison with authentic samples (Sigma^®^).

### 4.4. Animals

The in vivo assays were carried out in BALB/c male mice aged 8–10 weeks (25 ± 5 g) with glucose level values 150 ± 10 mg/dL. For the ex vivo assays, the organs were obtained from Sprague–Dawley male rats aged 16–20 weeks (450 ± 50 g). The animals were obtained from the Animal House of Centro Médico Nacional “Siglo XXI” at Instituto Mexicano del Seguro Social (IMSS). The mice were maintained at room temperature (22 ± 2 °C) on a 12 h light-dark natural cycle and fed with standard diet and water ad libitum. All investigations using experimental animals were conducted in accordance with the Official Mexican COM0062-ZOO-1999 [54] for Animal Experimentation and Care. All investigations were conducted with the approval of the Specialty Hospital Ethical Committee of Centro Médico Nacional “Siglo XXI” at IMSS (register: R-2015-3601-211 and R-2019-3601-004).

### 4.5. In Vivo Assays

#### 4.5.1. Induction of Experimental Type 2 Diabetes

The experimental diabetes mellitus was induced according to the streptozocin-induced type 2 (SID2) model described by Valdes et al. [12]. Mice were fasted for 16 h before receiving treatment (day 0). Streptozocin (STZ) was dissolved in a cold pH 4 buffer solution, then it was administered at 100 mg/kg intraperitoneally (IP) on days 1 and 3. Nicotinamide (NA) was dissolved in a cold saline solution and administered at 240 mg/kg IP 30 min after STZ treatment only on day 1. At the end of the treatment on day 3, a 10% sucrose solution was used ad libitum over two days. On day 5, the sucrose solution was withdrawn and substituted with water ad libitum. Then, 24 h later, the development of SID2 was determined by measuring postprandial blood glucose levels using a conventional glucometer (ACCU-CHECK^®^ Performa Blood Glucose Systems, Roche^®^, DC, Basel, Switzerland). Additionally, to confirm the SID2 model, β-cell function was evaluated with the administration of 5 mg/kg glibenclamide orally and measuring the decrease in glucose values 2 and 4 h after administration [36,55].

#### 4.5.2. Acute Antihyperglycemic Activity of Ethanolic Extract from *A. diversifolia* and Its Products

The procedure was conducted according to Calzada et al. [36]. Mice were randomly divided in 26 groups (*n* = 6 per group) as follows: normoglycemic mice control (NM) and SID2 control, both treated with vehicle (2% Tween 80 in water); 12 groups of NM; and 12 groups of SID2 treated with EEAd, CHCl_3_Fr, AcRFr, EtOAcFr (200 mg/kg), Fr5, farnesal, and farnesol (50 mg/kg). To compare the antihyperglycemic activities of the products isolated from *A. diversifolia*, the standard medications acarbose, canagliflozin, glibenclamide, pioglitazone, and metformin (50 mg/kg) were used. All treatments were solubilized in 2% Tween 80 in water and administered orally at 0.5 mL per mouse. The blood samples were collected from the tail vein before 0, 2 and 4 h after administration.

#### 4.5.3. Oral Sucrose and Lactose Tolerance Test of Ethanolic Extract from *A. diversifolia* and Its Products

OSTT was conducted according to Valdes et al. [12] in normoglycemic fasted male mice (FNM), which were randomly divided into eight groups (*n* = 6), as follows: vehicle group treated with 2% Tween 80 in water; sucrose group treated with vehicle + sucrose at 3 g/kg; five groups treated with EEAd, CHCl_3_Fr (200 mg/kg), Fr5, farnesal, and farnesol (50 mg/kg); and a group treated with acarbose (50 mg/kg), an α-glucosidase inhibitor, used as a pharmacological control. All treatments were solubilized in the vehicle and administered orally. Time 0 h was set before treatments; 30 min after the treatment, a sucrose load (3 g/kg) was administered to the mice. Blood samples were obtained 2 and 4 h after the administration of the carbohydrate using the glucose oxidase method [12,33]. The OLTT was performed under the same conditions as the OSTT assay, but in this case, a lactose load (3 g/kg) was given to the groups.

#### 4.5.4. Oral Glucose Tolerance Test of Ethanolic Extract from *A. diversifolia* and Its Products

OGTT assay was performed under the same conditions as the OSTT assay, but in this case, a glucose load (1.5 g/kg) was given to the groups, and canagliflozin (50 mg/kg), a moderate SGLT1 inhibitor, was used as a pharmacological control. The blood glucose measurements were recorded following the same method for the OGTT.

### 4.6. Ex Vivo Assays

#### 4.6.1. Intestinal Sucrose Hydrolysis Inhibition Assay of Ethanolic Extract from *A. diversifolia* and Its Products

The ISH assay was conducted in male Sprague–Dawley rats, which were sacrificed according to NOM0062-ZOO-1999. The proximal small intestine (SI) was removed and the first portions of the SI (jejunum and duodenum) were cut into 3 cm portions. These portions were tied on their ends with a non-absorbable silk suture (ETHICON^®^, Johnson & Johnson, Somerville, MA, USA). The group treatments (*n* = 6) were prepared as follows: EEAd, CHCl_3_Fr, and Fr5 (200, 400 and 800 μg/mL), and farnesol, farnesal, and acarbose (200, 400 and 800 μM). The samples were dissolved in 1.5 mL of 15% sucrose solution as a vehicle. Additionally, a control group was treated only with the vehicle. All the treatments were injected with an insulin syringe inside the 3 cm SI portions previously made in a 0.5 mL volume. Immediately, SI portions were placed in a Petri dish with 15 mL of distilled water as the external aqueous medium (EAM) and incubated during 2 h at 37 °C with constant agitation. The quantities of sucrose hydrolyzed and absorbed in the SI as glucose were measured in the EAM 2 h after adding the treatments using glucose oxidase method. The results were compared and normalized with the control group at the different measurement times and with the results of calculated half maximal effective concentration (CE_50_).

#### 4.6.2. Intestinal Glucose Absorption Inhibition Assay of Ethanolic Extract from *A. diversifolia* and Its Products

The IGA assay was conducted under similar conditions to the ISH test. The only difference was that in the IGA assay, a 5% glucose solution was used as the vehicle. The pharmacological control was canagliflozin (200, 400 and 800 μM) and the measurement was recorded 1 h after adding the treatments. In this test, the measurement times changed due to the use of a simple carbohydrate, which does not need to be hydrolyzed to be absorbed; thus, its absorption is instant.

### 4.7. Urinary Glucose Excretion Assay of Ethanolic Extract from A. diversifolia and Its Products

The UGE test was conducted on male normoglycemic BALB/c mice, which were randomly divided into 7 groups (*n* = 6) as follows: one control group treated with vehicle (2% Tween 80 in water), six groups treated with EEAd, CHCl_3_Fr (200 mg/kg), Fr5, farnesal, farnesol, or canagliflozin as a pharmacological control (50 mg/kg). All treatments were solubilized in the vehicle and administered orally at 0.5 mL volume per mice. After the treatments, animals were placed in a metabolic cage for 2 h with free access to water. During the 2 h, the urine was collected, and the glucose concentration (mg/dL) in urine was determined using a conventional glucometer (ACCU-CHECK^®^ Performa Blood Glucose Systems, Roche^®^).

### 4.8. Statistical Analysis

All the results are expressed as mean values ± standard error of the mean (SEM). All statistical analyses were performed using GraphPad Prism version 8 for Macintosh (GraphPad Software Inc., San Diego, CA, USA). The statistical evaluation was conducted through an analysis of variance followed by a Bonferroni test for multiple comparisons. *p* < 0.05 was considered a statistically significant difference between the group means.

## 5. Conclusions

The complete analysis of results suggested that control of hyperglycemia after administration of *A. diversifolia* and its products is mediated by the inhibition of hydrolysis of complex disaccharides and absorption of simple monosaccharides through inhibition of α-glucosidase and selective SGLT-1 inhibition in the small intestine. However, more studies are needed to confirm both mechanisms of action. In addition, the results validate the use of *A. diversifolia* in Mexican traditional medicine for the treatment of diabetes. 

## Figures and Tables

**Table 1:** Blood glucose levels of male normoglycemic mice (NM) and streptozocin-induced type 2 diabetes mice (SID2) at 0, 2 and 4 h on the acute antihyperglycemic test.

Treatment	Glycemia (mg/dL)		
	0 h	2 h	4 h
**NM Control**	137.3 ± 4.7	131.8 ± 2.4	134.8 ± 2.1
**NM + EEAd**	138.7 ± 8.5	122 ± 6.6	128 ± 9
**NM + CHCl_3_Fr**	142 ± 10.9	133.7 ± 4.9	124.7 ± 4
**NM + AcRFr**	141 ± 5.4	167.7 ± 7.3 *^,⬪^	175.3 ± 10.2 *^,⬪⬪^
**NM + EtOAcFr**	141 ± 2	171 ± 8.1 *^,⬪^	167.3 ± 5.1 *^,⬪⬪^
**NM + Fr5**	148.3 ± 4	134.1 ± 4.8	145.7 ± 10.9
**NM + Farnesal**	143.7 ± 6.1	133.7 ± 3.6	128.3 ± 2.9
**NM + Farnesol**	136 ± 6.6	130.7 ± 5.2	128 ± 5.5
**NM + Acarbose**	137.7 ± 3.6	130.7 ± 2.3	125.3 ± 2.9
**NM + Canagliflozin**	140.8 ± 2.9	106.8 ± 2.2 *^,⬪^	95.2 ± 0.4 *^,⬪⬪^
**NM + Glibenclamide**	144.7 ± 2.6	95.7 ± 1.1 *^,⬪^	84.6 ± 2.8 *^,⬪⬪^
**NM + Pioglitazone**	140.3 ± 3.8	109 ± 1.9 *^,⬪^	123.7 ± 1.3 *
**NM + Metformin**	139.3 ± 1.3	125 ± 3.4	142 ± 0.8
**SID2 Control**	331 ± 14.4	353.7 ± 5.7	336 ± 2.9
**SID2 + EEAd**	366.3 ± 18.4	221 ± 35.5 *^,Ψ^	324 ± 17.9
**SID2 + CHCl_3_Fr**	336.7 ± 21.6	230.3 ± 13.3 *^,Ψ^	287.7 ± 5.6 *^,^^ΨΨ^
**SID2 + AcRFr**	354.3 ± 12.4	398 ± 23.5 *^,Ψ^	439 ± 14.2 *^,ΨΨ^
**SID2 + EtOAcFr**	337.8 ± 3.6	377 ± 3.1 *^,Ψ^	365.5 ± 11.1 *^,ΨΨ^
**SID2 + Fr5**	350.7 ± 26	213.5 ± 35.5 *^,Ψ^	209 ± 12.6 *^,ΨΨ^
**SID2 + Farnesal**	337.3 ± 13.1	268 ± 12.2 *^,Ψ^	226 ± 8 *^,ΨΨ^
**SID2 + Farnesol**	339.7 ± 10.3	255.3 ± 23.1 *^,Ψ^	337 ± 7.2
**SID2 + Acarbose**	335.8 ± 8.7	255.5 ± 20.5 *^,Ψ^	323.3 ± 11.6
**SID2 + Canagliflozin**	367.3 ± 8.4	157.7 ± 31.5 *^,Ψ^	102 ± 11.5 *^,ΨΨ^
**SID2 + Glibenclamide**	357 ± 7.5	254.3 ± 3.3 *^,Ψ^	234.3 ± 19.3 *^,ΨΨ^
**SID2 + Pioglitazone**	335.7 ± 8.2	259.7 ± 7.9 *^,Ψ^	248.3 ± 4.4 *^,ΨΨ^
**SID2 + Metformin**	338.7 ± 13.5	275 ± 7.6 *	266.7 ± 7.2 *^,ΨΨ^

EEAd, CHCl_3_Fr, AcRFr, and EtOAcFr were administered at 200 mg/kg; Fr5, farnesal farnesol, acarbose, canagliflozin, glibenclamide, pioglitazone and metrformin were administered at 50 mg/kg. Data are expressed as means ± SEM, *n* = 6; * *p* < 0.05 vs. initial values; ^⬪^ *p* < 0.05 vs. NM control for 2 h; ^⬪⬪^ *p* < 0.05 vs. NM control for 4 h; ^Ψ^ *p* < 0.05 vs. SID2 control for 2 h; ^ΨΨ^ *p* < 0.05 vs. SID2 control for 4 h. SEM: standard error of the mean; SID2: streptozocin-induced type 2 diabetes mice. Acarbose, canagliflozin, glibenclamide, and pioglitazone were used as pharmacological controls.

**Table 2:** Quantity of glucose measured in the external aqueous medium, percent of inhibition and CE_50_ calculated after addition of treatments on intestinal sucrose hydrolysis (ISH) inhibition test.

Treatment	Glucose (mg/dL)	Glucose (mg/dL)	% of Inhibition	CE_50_
	0 h	2 h		
**Sucrose (15%)**	0 ± 0	89.3 ± 17.5	-	-
**EEAd [200 µg/mL]**	0 ± 0	63.6 ± 7.5 *	28.70	565.6 µg/mL
**EEAd [400 µg/mL]**	0 ± 0	52.33 ± 10 *	41.39	
**EEAd [800 µg/mL]**	0 ± 0	33 ± 4 *	63.04	
**CHCl_3_Fr [200 µg/mL]**	0 ± 0	107 ± 6.4	0	662.2 µg/mL
**CHCl_3_Fr [400 µg/mL]**	0 ± 0	65.6 ± 2.9 *	26.46	
**CHCl_3_Fr [800 µg/mL]**	0 ± 0	33 ± 7.6 *	63.04	
**Fr 5 [200 µg/mL]**	0 ± 0	85.3 ± 5.8	4.41	590.4 µg/mL
**Fr 5 [400 µg/mL]**	0 ± 0	59 ± 4.4 *	33.93	
**Fr 5 [800 µg/mL]**	0 ± 0	25.66 ± 2.1 *	71.25	
**Farnesal [200 µM]**	0 ± 0	93 ± 9.8	0	682.9 µM
**Farnesal [400 µM**	0 ± 0	64.6 ± 8.8	27.58	
**Farnesal [800 µM]**	0 ± 0	36 ± 2.5 *	59.68	
**Farnesol [200 µM]**	0 ± 0	84.33 ± 2.6	5.56	802.2 µM
**Farnesol [400 µM]**	0 ± 0	67.33 ± 1.76	24.59	
**Farnesol [800 µM]**	0 ± 0	45.6 ± 1.2 *	48.86	
**Acarbose [200 µM]**	0 ± 0	36.3 ± 4 *	59.31	187.8 µM
**Acarbose [400 µM]**	0 ± 0	11.33 ± 0.89 *	87.30	
**Acarbose [800 µM]**	0 ± 0	6.66 ± 0.33 *	90.29	

Quantity of glucose absorbed and measured in external aqueous medium (mg/dL) expressed as means ± SEM, *n* = 6; * *p* < 0.05 vs. Sucrose (15%) group for 2 h. CE_50_: half maximal effective concentration.

**Table 3:** Quantity of glucose measured in the external aqueous medium, percent inhibition, and CE_50_ calculated after addition of treatments on intestinal glucose hydrolysis (IGA) inhibition test.

Treatment	Glucose (mg/dL)	Glucose (mg/dL)	% of Inhibition	CE_50_
	0 h	1 h		
**Glucose (5%)**	0 ± 0	217.3 ± 8.7	-	-
**EEAd [200 µg/mL]**	0 ± 0	202.6 ± 5.2	6.73	1059.9 µg/mL
**EEAd [400 µg/mL]**	0 ± 0	162.3 ± 30	25.29	
**EEAd [800 µg/mL]**	0 ± 0	138.6 ± 5.2 *	36.18	
**CHCl_3_Fr [200 µg/mL]**	0 ± 0	308.6 ± 25.9	0	783.5 µg/mL
**CHCl_3_Fr [400 µg/mL]**	0 ± 0	182.3 ± 6.2 *	16.09	
**CHCl_3_Fr [800 µg/mL]**	0 ± 0	105 ± 2.8 *	51.67	
**Fr 5 [200 µg/mL]**	0 ± 0	255.6 ± 11.7	0	539.9 µg/mL
**Fr 5 [400 µg/mL]**	0 ± 0	114 ± 7.7 *	47.57	
**Fr 5 [800 µg/mL]**	0 ± 0	51 ± 1.52 *	76.53	
**Farnesal [200 µM]**	0 ± 0	311 ± 11.06	0	1211.8 µM
**Farnesal [400 µM]**	0 ± 0	255.3 ± 14	6.46	
**Farnesal [800 µM]**	0 ± 0	152 ± 35.6 *	30.05	
**Farnesol [200 µM]**	0 ± 0	398.6 ± 39.8	0	372.3 µM
**Farnesol [400 µM]**	0 ± 0	86.3 ± 20.5 *	60.26	
**Farnesol [800 µM]**	0 ± 0	112.3 ± 20.6 *	48.30	
**Canagliflozin [200 µM]**	0 ± 0	237.6 ± 22	0	763.0 µM
**Canagliflozin [400 µM]**	0 ± 0	112.3 ± 14.1 *	48.30	
**Canagliflozin [800 µM]**	0 ± 0	119 ± 8.5 *	45.23	

Quantity of glucose absorbed and measured in external aqueous medium (mg/dL) expressed as means ± SEM, *n* = 6; * *p* < 0.05 vs. Glucose (5%) group for 1 h. CE_50_: half maximal effective concentration.

**Table 4:** ^1^H and ^13^C (700 MHz) NMR data of compounds **1** (farnesal) and **2** (farnesol) in CDCl_3_.

Position	Farnesal (1)		Farnesol (2)	
	δ_H_, mult. (*J* in Hz)	δ_C_, Type	δ_H_ mult. (*J* in Hz)	δ_C_, Type
**1**	9.97, d (8.04)	59.14, CH	4.1, *d* (8)	59.14, CH_2_
**2**	5.86, dd, (8.1, 4.3)	124.5, CH	5.37, *t* (8)	124.5, CH
**3**	-	136.18, C	-	136.18, C
**4**	2.01, t (3.2)	35.27, CH_2_	1.97, *s*	35.27, CH_2_
**5**	2.15, t (1.44)	32.35, CH_2_	1.97, *s*	32.35, CH_2_
**6**	5.07, s	124.66, CH	5.4, *s*	124.66, CH
**7**	-	135.48, C	-	135.48, C
**8**	2.15, t (1.44)	28.11, CH_2_	1.97, *s*	28.11, CH_2_
**9**	2.15, t (1.44)	26.54, CH_2_	1.97, *s*	26.54, CH_2_
**10**	5.07, s	125.14, CH	5.1, *s*	125.14, CH
**11**	-	135.36, C	-	135.36, C
**12**	1.58, d (3.49)	16.13, CH_3_	1.55, *s*	16.13, CH_3_
**13**	1.96, t (1.58)	25.82, CH_3_	1.68, *s*	25.82, CH_3_
**14**	1.66, d (5.51)	23.57, CH_3_	1.68, *s*	23.57, CH_3_
**15**	1.58, d (3.49)	19.89, CH_3_	1.68, *s*	19.89, CH_3_
